# Safety Study on Ketoprofen in Pigs: Evaluating the Effects of Different Dosing and Treatment Scheme on Hematological, Hepatic, and Renal Parameters

**DOI:** 10.3390/vetsci8020030

**Published:** 2021-02-18

**Authors:** Luis Emilio Fazzio, Santiago José Raggio, Juan Facundo Romero, Juver Membrebe, Antonio Humberto Hamad Minervino

**Affiliations:** 1 Laboratorio de Patología Especial Veterinaria, Veterinary Faculty, La Plata University, Avenue 60 and 118 s/n, La Plata 1900, Argentina; 2 Biogenésis Bagó, Panamericana Km 38,5, Garín, Buenos Aires B1619, Argentina; santiago.raggio@biogenesisbago.com; 3 Atención Veterinaria Integral Chascomús (AVICh) Ensayos Clínicos Veterinarios, Cinacinas 105, Chascomús, Buenos Aires CP 7130, Argentina; juanfacundoromero@yahoo.com.ar; 4 Biogenésis Bagó (Shanghai) Biotechnology Company Limited., 828-838 Zhangyang Road, Huadu Mansion Pudong New District, Shanghai 200122, China; juver.membrebe@biogenesisbago.com; 5 Laboratory of Animal Health, LARSANA, Federal University of Western Pará, UFOPA, Santarém 68040-080, Brazil

**Keywords:** non-steroidal anti-inflammatory drug, intramuscular treatment, safety, swine

## Abstract

A safety study on ketoprofen 10% was carried out on pigs using a different dosing and treatment scheme. Forty healthy crossbreed pigs with similar age, weight, and body condition score were distributed into five treatment groups. The pigs were intramuscularly injected once with different doses of ketoprofen: 3 mg/kg (group 1X), 6 mg/kg (group 2X), 9 mg/kg (group 3X). In addition, the 3 mg/kg dosis was administered on three consecutive days (group 1X ext.). Intramuscular injections of saline solution were used in control group (CTL). The pigs were clinically examined throughout the trial and blood samples were taken for hematological and biochemical evaluation on days −4 (before treatment), +3, +7, and +14 (the end of the trial). Any unusual behaviour or clinical signs were reported as potential toxic effects of ketoprofen. Serum measurements showed that none of the ketoprofen doses produced changes in renal or hepatic biochemical parameters, liver enzymes, or total bilirubin. Likewise, hematological assessment indicated no altered parameters or hematocrit percentage in the study groups. These results demonstrate that ketoprofen has no adverse effects in pigs when the doses and scheme evaluated in this study are applied.

## 1. Introduction

Ketoprofen is a non-steroidal anti-inflammatory drug (NSAID) that derives from propionic acid [1]. These drugs act through the non-selective inhibition of the two cyclooxygenase isoforms (COX1 and COX2). Cyclooxygensase-1 produces toxic effects, such as altered gastrointestinal function, hepatopathies, nephrotoxicity, and coagulation disorders [2,3]. In contrast, COX2 inhibition exerts NSAID therapeutic effects [4,5,6].

Ketoprofen is a chiral compound that contains one asymmetric carbon atom and thus exists in two enantiomeric forms (R and S ketoprofen) [2,7] which are detected in plasma after intramuscular injections [3,8,9]. Ketoprofen is then inactivated in the liver through hydrolysis and glucuronic acid conjugation [10].

In veterinary medicine, ketoprofen is used as an antipyretic, anti-inflammatory, and analgesic agent [3,11,12,13,14]. A dose of 3 mg/kg reduced mortality rates in pre-weaning piglets [15], improved body conditions of postpartum sows [16], was effective in treating sows with lameness [3] and reduced body temperature in pigs challenged with *Actinobacillus pleuropneumoniae* [11], but was ineffective at alleviating surgical castration pain in piglets [17].

Although there are several studies on ketoprofen in many other species, published data on pigs are scarce [5]. Its potential adverse effects on the indicators of kidney damage (urea and creatinine), liver enzyme function (alkaline phosphatase, aspartate aminotransferase, gamma glutamyl transpeptidase, total bilirubin), and hematological parameters (erythrocytes, leukocytes, platelets) have not been evaluated when using different doses and treatment schemes. The aim of this work was to evaluate the impact of ketoprofen on hematological, hepatic and renal parameters using a different dosing and treatment scheme in pigs.

## 2. Materials and Methods

All the experimental procedures were approved by the Institutional Animal Care and Use Committee (CICUAL, for its Spanish acronym), Faculty of Veterinary Sciences, National University of La Plata, Argentina (Protocol No 107-1-20P).

This trial was performed on a commercial farm located in the district of Castelli, Buenos Aires Province, Argentina (−36.176225, −57.762943). The farm has a capacity for 250 sows on a single site positive for enzootic pneumonia/swine influenza and negative for PRRS. The pigs were managed as per usual practice on the farm. Forty crossbreed pigs (Duroc Jersey x Landrace x Yorkshire) of both sexes were selected (17 males; 23 females). They were clinically healthy and had similar age (60 ± 3 days), body weight (22.4 ± 3.2 kg), and body condition score 4 (1–5 scale). All the animals were identified with a tag in the left ear. Weight and body condition scores were individually evaluated before ketoprofen administration (day –4 of the trial). The selected pigs were grouped according to their sex and weight and then randomly distributed into five treatment groups: group 1X (3 mg/kg ketoprofen, 1 mL/33 kg BW; Ketoprofeno Biogénesis Bagó^®^, Biogénesis Bagó, Buenos Aires, Argentina); group 2X (6 mg/kg ketoprofen, 2 mL/33 kg BW); group 3X (9 mg/kg ketoprofen, 3 mL/33 kg BW), group 1X ext. (extended, 3 mg/kg ketoprofen, 1 mL/33 kg BW administered on 3 consecutive days within a 24 h interval) and CTL group (sterile saline solution, 1 mL/33 kg BW). The ketoprofen and saline solution were administered via intramuscular route. Day 0 corresponded to ketoprofen or sterile saline solution treatment day for 1X, 2X, 3X and CTL groups. In 1X ext. group, the first, second and third dose was given on day –2, day –1 and day 0, respectively. All pigs were examined daily throughout the trial (from day −4 to day +14). Behavior, manifestation, of pain and local inflammation at the injection site were also evaluated.

Blood samples with anticoagulant (EDTA) and without anticoagulant were collected via jugular venipuncture on days −4, +3, +7 and +14. The blood samples without anticoagulant were transported to the laboratory on the farm to be centrifuged (3000 rpm, 10 min). The resultant sera were stored at −20 °C until their analysis. Samples with EDTA were processed on sampling day. Erythrocyte count, total leukocyte count, platelet count, hematimetric indices, and hematocrit percentage were obtained by using a veterinary blood counter (Model BC2800, Mindray Medical International Limited, Shenzhen, China). The hematocrit percentage and platelet count were also checked through a manual microhematocrit technique and microscopy visualization (Giemsa staining), respectively.

Serum biochemical parameters were estimated with a standard assay kit (Wiener Laboratories, Rosario, Argentina) and processed using a clinical chemistry analyzer (Mindray BS300, Shenzhen, China). Urease method, Jaffe’s kinetic method, DGKC optimized kinetic method, IFCC kinetic method and DPD method were used to measure urea, creatinine, alkaline phosphatase, aspartate aminotransferase/gamma-glutamyl transpeptidase, and total bilirubin concentrations, respectively. Kidney function was monitored by means of urea and creatinine concentrations whereas liver function was assessed through serum alkaline phosphatase, aspartate aminotransferase, gamma-glutamyl transpeptidase, and total bilirubin concentrations.

A completely randomized design was used. The data were analyzed using a mixed model with repeated measures over time with the Statistical Analysis System (SAS) statistical software (9.1). The fixed variables were group (1X vs. 2X vs. 3X vs. 1X ext. vs. CTL), time (day −4 vs. +3 vs. +7 vs. +14) and their interaction (group-by-time). The animal within each treatment was the random variable. SLICE option of SAS was used to detect means when differences were significant. Significance was established as *p* < 0.05 for the main effects and for their interaction.

## 3. Results and Discussion

Unusual behaviour such as depression, ataxia or prostration was not reported as a potential sign of toxicity associated with ketoprofen doses (1X, 2X, 3X) and treatment scheme (1X ext.) throughout this study. No local inflammation or pain was observed at the injection site.

The main evaluated blood components are presented in Table 1. There were no time interactions or differences in hematocrit percentage, erythrocyte count, total leukocyte count and platelet count among the groups. The obtained values were within a normal range and no treatment-driven changes were reported. Hematimetric indices and hemoglobin concentration were similar in all the groups without group-by-time interactions (*p* > 0.1). Other authors observed thrombocytopenia after ketoprofen use in humans and dogs as a result of lactate dehydrogenase reduction in platelets is reduced [18,19]. However, the platelet counts reported in this study were within a normal range indicating that hematological parameters were not altered by the evaluated ketoprofen doses and treatment scheme.

Liver function assessed through serum concentrations of different enzymes and bilirubin is shown in Table 2. All samplings indicated no changes due to ketoprofen administration. The mean values of alkaline phosphatase were above the normal range at the beginning of the treatment. Although its post-treatment values remained high, they were kept barely above the normal range. This enzyme is used as a marker of cholestasis in most animals [20]. Even though it is particularly concentrated in the hepatocellular cytoplasm, it is present in different body tissues. Thus, its activity should be considered together with other physiological factors such as age, nutritional status and heat stress [20,21,22]. In this study, no alkaline phosphatase between-group variations or group-by-day interactions were reported, which indicates that its concentrations were not increased after ketoprofen use.

Moreover, aspartate aminotransferase and gamma-glutamyl transpeptidase concentrations were within a normal range without changes due to treatment. Aspartate aminotransferase is found in the mitochondria of liver cells whereas gamma-glutamyl transpeptidase is present in their cytoplasm [20]. The latter is also present in various body tissues and plays an important role in antioxidant mechanisms [23]. Consequently, its activity is also affected by oxidative stimuli in several species, including pigs [24]. Total bilirubin count is the sum of direct and indirect bilirubin. Indirect or non-conjugated bilirubin is pre-hepatic and increases when hemolysis occurs. Direct or conjugated bilirubin is a marker of cholestasis [20]. In this study, the total bilirubin concentration indicated no liver damage or intravascular hemolysis in this study. Mean values were within a normal range showing no statistically significant differences related to the treatment. Combined liver markers (alkaline phosphate, aspartate aminotransferase, gamma-glutamyl transpeptidase, and total bilirubin) showed normal liver function regardless of the dose and treatment scheme applied.

Kidney function evaluated according to urea and creatinine concentrations are shown in Table 3. All samplings in this study showed no related-to-treatment differences. When kidney failure occurs, both concentrations are above the normal range [25,26]. All samplings in this study showed no related-to-treatment differences. Therefore, they were indicative of preserved kidney function after treatment.

## 4. Conclusions

Ketoprofen administered intramuscularly in pigs at doses of 3, 6 and 9 mg/kg (groups 1X, 2X, and 3X, respectively) did not produce any changes in hematological, hepatic, and renal parameters. Furthermore, these parameters remained normal when ketoprofen was consecutively administered for three days at a dose of 3 mg/kg bodyweight. The present study demonstrated that the use of ketoprofen at a higher dose than that established by the manufacturer’s laboratory and/or at repeated doses does not induce alterations in the investigated parameters.

## Figures and Tables

**Table 1:** Least squares means for hematocrit percentage and erythrocyte, leukocyte and platelet counts in different groups treated with ketoprofen or sterile saline solution.

Day	Group (Least Squares Means)					*p* Value			
	1X	2X	3X	1X ext.	CTL	SEM	Day	Group	Day by Group
Hemotocrit (normal range 33–50%)									
−4	35.3	33.4	35.4	34.5	37.1	0.9	<0.001	0.1919	0.4499
3	31.5	32.4	33	31.4	33.9				
7	32.1	32.1	32.4	31.9	34.1				
14	32.9	31.8	34.1	34.1	34.9				
Erythrocytes (normal range 5–8 million/mm^3^)									
−4	6.2	6	6.4	6.4	6.5	0.17	<0.001	0.1228	0.4675
3	5.6	5.7	6	5.9	6				
7	5.9	5.9	6	6	6.3				
14	5.7	5.5	5.9	6.3	6.1				
Leukocytes (normal range 10–22 miles/mm^3^)									
−4	14.9	13.9	17.5	16.8	18.9	1.2	<0.001	0.2059	0.3308
3	14.6	13.3	21.3	15.7	15.8				
7	13	12.2	18.4	15.6	14.5				
14	11.7	12.4	13.4	14	12.4				
Platelets (normal range 200–800 miles/mm^3^)									
−4	475	443	435	455	411	4.5	0.006	0.8921	0.9234
3	453	487	461	499	430				
7	429	404	398	457	369				
14	415	371	428	393	400				

Group 1X (3 mg/kg ketoprofen, 1 mL/33 kg. BW, *n* = 8); Group 2X (6 mg/kg. de ketoprofen, 2 mL/33 kg. BW, *n* = 8); Group 3X (9 mg/kg. ketoprofen, 3 mL/33 kg. BW, *n* = 8), Group 1X-ext (3 mg/kg ketoprofen, 1 mL/33 kg. BW for 3 consecutive days with a 24 h interval, *n* = 8) and Control Group (sterile saline solution 1 mL/33 kg. BW, *n* = 8). Day –4 (4 days before treatment); day 0 (treatment day for group 1X, 2X, 3X and last dose in the extended group), day +3 (3 days after treatment), day +7 (7 days after treatment) and day +14 (14 days after treatment). SEM: standard error of the mean.

**Table 2:** Least squares means for alkaline phosphatase, aspartate aminotransferase, gamma-glutamyl transpeptidase, total bilirubin concentrations in different groups treated with ketoprofen or sterile saline solution.

Day	Group (Least Squares Means)					*p* Value			
	1X	2X	3X	1X ext.	CTL	SEM	Day	Group	Day by Group
Alkaline phosphatase (normal range 118–395 IU/L)									
–4	594.6	526.1	506.9	518.3	524.2	32	<0.001	0.43	0.89
+3	429.8	381.8	366.8	379.8	410.5				
+7	418.6	370	343.5	379.6	416				
+14	465.8	422.9	407.6	449.1	463.2				
Aspartate aminotransferase (normal range 9–113 IU/L)									
–4	30.0	33.4	31.8	27.8	31.4	16	0.06	0.36	0.67
+3	29.0	28.5	90.2	30.4	28.9				
+7	32.4	32.6	42.4	29	30.9				
+14	51.9	40.8	58.3	57.4	69.1				
Gamma glutamyl transpeptidase (normal range 10–60 IU/L)									
–4	34.6	31.8	21.6	28.6	34.1	3.7	<0.001	0.083	0.89
+3	32.1	28.4	19.5	27.9	33.4				
+7	33.3	31	19.9	28.6	33.5				
+14	40.1	36	28.4	36.5	37.9				
Total bilirubin (normal range 0.1–1 mg/100 mL)									
–4	0.16	0.27	0.19	0.16	0.19	0.06	0.002	0.18	0.33
+3	0.42	0.29	0.46	0.28	0.23				
+7	0.28	0.28	0.43	0.21	0.29				
+14	0.32	0.24	0.29	0.27	0.29				

Group 1X (3 mg/kg ketoprofen, 1 mL/33 kg. BW, *n* = 8); Group 2X (6 mg/kg. de ketoprofen, 2 mL/33 kg. BW, *n* = 8); Group 3X (9 mg/kg. ketoprofen, 3 mL/33 kg. BW, *n* = 8), Group 1X-ext (3 mg/kg ketoprofen, 1 mL/33 kg. BW for 3 consecutive days with a 24-h interval, *n* = 8) and Control Group (sterile saline solution 1 mL/33 kg. BW, *n* = 8). Day –4 (4 days before treatment); day 0 (treatment day for group 1X, 2X, 3X and last dose in the extended group), day +3 (3 days after treatment), day +7 (7 days after treatment) and day +14 (14 days after treatment). IU/L: international units per litre. SEM: standard error of the mean.

**Table 3:** Least squares means for urea and creatinine concentrations in different groups treated with ketoprofen or sterile saline solution.

Day	Group (Least Squares Means)					*p* Value			
	1X	2X	3X	1X ext.	CTL	SEM	Day	Group	Day by Group
Urea (normal range 21–64 mg%)									
–4	17.9	18.4	16.8	15.8	14.4	1.77	<0.001	0.426	0.471
+3	14.4	18.9	14.1	15.0	14.8				
+7	20.1	19.5	17.3	18.4	19.0				
+14	25.3	27.4	23.4	27.1	23.4				
Creatinine (normal range 1–2.7 mg%)									
–4	1.0	1.0	1.0	1.0	1.1	0.03	<0.001	0.504	0.370
+3	0.9	1.0	1.0	0.9	1.0				
+7	1.0	1.0	1.0	0.9	1.0				
+14	1.1	1.1	1.1	1.1	1.1				

Group 1X (3 mg/kg ketoprofen, 1 mL/33 kg. BW, *n* = 8); Group 2X (6 mg/kg. de ketoprofen, 2 mL/33 kg. BW, *n* = 8); Group 3X (9 mg/kg. ketoprofen, 3 mL/33 kg. BW, *n* = 8), Group 1X-ext (3 mg/kg ketoprofen, 1 mL/33 kg. BW for 3 consecutive days with a 24-h interval, *n* = 8) and Control Group (sterile saline solution 1 mL/33 kg. BW, *n* = 8). Day –4 (4 days before treatment); day 0 (treatment day for group 1X, 2X, 3X and last dose in the extended group), day +3 (3 days after treatment), day +7 (7 days after treatment) and day +14 (14 days after treatment). SEM: standard error of the mean.

## Data Availability

The raw data regarding this article is available upon request to the corresponding author.
